# Analysis of blood gases, serum fat and serum protein: a new approach to estimate survival chances of stranded Harbor seal (*Phoca vitulina*) pups from the German North Sea

**DOI:** 10.1186/1751-0147-56-10

**Published:** 2014-02-04

**Authors:** Katharina A Witte, Jörg Driver, Tanja Rosenberger, Sven Adler, Ursula Siebert

**Affiliations:** 1 Institute of Terrestrial and Aquatic Wildlife Research (ITAW), University of Veterinary Medicine Hannover, Foundation, Werftstrasse 6, Büsum D-25761, Germany; 2 Veterinary Clinic, Bosselweg 10, Reinsbüttel D-25764, Germany; 3 Seal Center Friedrichskoog, An der Seeschleuse 4, Friedrichskoog D-25718, Germany; 4 Swedish University of Agricultural Sciences, Umeå 901 83, Sweden

**Keywords:** Blood gas analysis, Serum fat (triglycerides), Serum protein, Harbor seal pups, Rehabilitation, *Phoca vitulina*

## Abstract

**Background:**

Facing numerous challenges, such as illness, storms or human disturbance, some harbor seal (*Phoca vitulina*) pups lose contact to their dams and are found abandoned along the North Sea coast. In Schleswig-Holstein, pups with the prospect of surviving rehabilitation are admitted to the Seal Center Friedrichskoog. Despite elaborate clinical health assessments on admission, including differential hematology, in 2010, 17% of 108 admitted pups did not survive the first 20 days. The death rate during the years 2006 and 2009 varied between 9 and 19%. To broaden the spectrum of variables which could be predictive for survival, blood gas and serum analyses were performed for 99 pups using venous blood. Variables included total CO_2_, pH, partial CO_2_, HCO_3_^–^, base excess and anion gap as well as glucose, urea nitrogen, sodium, potassium and chloride. Moreover, total serum protein and fat (triglyceride) concentrations were measured for all pups on admission.

**Results:**

Repeated measurements of 12 randomly selected individuals revealed a significant (p = 0.002) positive influence of time in rehabilitation on triglyceride concentrations. This trend probably shows the improvement of the pups’ nutritional status as a consequence of the shift from milk replacer formula to fish. No such positive influence was detected for total protein concentrations though. Hematologic values, including blood gases, were not predictive for survival.

**Conclusions:**

For the first time blood gas values are reported in this study for a large sample size (N = 99) of seal pups (regardless of their health status). The ranges and medians calculated from the data can serve as a stepping stone towards the establishment of reference values for neonate harbor seals. However, future investigations on the development of blood gases in harbor seals with different health conditions and ages over time are necessary to allow for a better understanding of acid–base regulation in harbor seals.

## Introduction

One of the three indigenous marine mammals in the German North Sea is the harbor seal (*Phoca vitulina*). Its pupping season in the Wadden Sea area of Schleswig-Holstein, Germany, usually lasts from late May to mid-July. The majority of pups (95%) are born in June. However, the first pups are occasionally born in early May [1-3].

Lactation lasts for 4 weeks with a milk containing up to 50% fat [4] and takes place on sandbanks and beaches in tidal areas during low tide [1,2,5]. Although pups are born fully developed and capable of swimming and diving with their mothers within hours after birth, around 30% of pups die during the first year after birth, mainly because of illness or separation from their mothers [5]. Some pups are born prematurely and therefore not fully developed or still carrying their lanugo fur, which is normally shed in utero. Summer storms, the death of the mother, or weakness of the pup can lead to the separation of pups from their mothers [6,7]. While it is assumed that pups attend their mothers’ foraging trips separation is possible as trips can last 7 to 10 hours and weaken pups [4].

According to Brasseur & Fedak [5], anthropogenic disturbances might cause pups to miss suckling through a whole tide resulting in a deficit of 50% of their daily caloric intake and cause dehydration [8]. Frequent disturbances may affect the pups’ total weaning masses and thus their survival probability and can also be a reason for the separation of mother and pup. According to Brasseur & Fedak [5], the high youth mortality in the Wadden Sea (30-35% vs. 25% elders) might be an indicator for a high anthropogenic disturbance level.

For these natural and anthropogenic reasons unweaned pups are sometimes found abandoned along beaches of the German North Sea coast and admitted to the seal center when having a normal blood status [6]. During the first weeks of rehabilitation pups die due to septicemia, anorexia, gastroenteritis and fetal atelectasis (Siebert pers. comm).

Although the harbor seal population of the North Sea is not at risk of extinction, governments of coastal states are responsible for the management and supervision of this wild population. In Germany, stranded animals in good condition with the perspective of survival are admitted to a rehabilitation facility. However, numbers of removed and reintroduced animals should be kept at a minimum. Rehabilitation should not last longer than a few months, and pups should be in good health before being reintroduced into the wild [3].

Certain components of blood reflect the balance (homeostasis) of nutrients, storage of water and proteins (e.g. urea, proteins, fats, glucose) [9,10]. The latter were investigated thoroughly in free-ranging, rehabilitated and captive harbor seals of different age classes in the German North Sea by Hasselmeier *et al.*[7].

Blood gases are involved in breathing and metabolic processes of vertebrates. The dispersal of gases (partial pressure) such as O_2_ and CO_2_ (and its derivatives) is essential for maintaining a steady pH, ion balance and acid–base balance. Numerous investigations on the respiratory component of blood gases in connection with diving physiology of harbor seals have been conducted (e.g. [11-13]). However, little is known concerning the metabolic component of the acid–base balance including pH, bicarbonate and carbon dioxide concentrations in harbor seals. Arterial blood is the preferred medium for the assessment of acid–base imbalances related to respiratory dysfunctions, because it is quite homogeneously in its gas composition despite the area from where it is withdrawn while venous blood is used to assess electrolytes and metabolic dysfunctions [14]. Furthermore, the present study focused on metabolic dysfunctions and therefore venous blood was the preferred medium.

Different variables and indicators can help detect acid–base disorders and their characteristics (alkalosis or acidosis of respiratory or metabolic origin). Nomograms [15] or oxygen status algorithms [16] can provide information on imbalances, while single variables such as anion gap or base excess of the extracellular fluid (BE^ecf^) can provide hints of the potential source of imbalance [17,18].

Another helpful indicator for the assessment of the health status of harbor seal pups is the calculation of a body condition index. There are numerous equations for the calculation of the body condition index that are suitable for harbor and gray seals (e.g. [19-23]). First year survival was found to be correlated with weaning mass in gray seals (*Halichoerus grypus*) by Hall *et al.*[23], and autumnal mass was linked to over-winter survival in harbor seals by Harding *et al.*[24].

Blood gas variables have not been measured before on a comparably high number of harbor seals, not to mention neonate harbor seals. If at all, only single variables and no complete sets were measured, using different analyzing techniques [25-27].

In this study, the set of variables usually measured in hemogram profiles was supplemented by the analysis of serum chemistry variables (glucose, urea nitrogen, total serum protein and triglycerides), electrolytes (Na^+^, K^+^, Cl^-^ and anion gap) and blood gases (TCO_2_, pH, pCO_2_, HCO_3_^–^ and base excess (BE^ecf^)) to develop further indicators for the estimation of the health status and survival chances of harbor seal pups during the first weeks of rehabilitation. It is assumed that a majority of pups show low triglyceride concentrations on admission to rehabilitation due to emaciation before being found. Furthermore, we expect an increase of this parameter over time, especially after the shift from milk replacer formula to fish. The overall purpose of this study is to have additional clinical parameters to recognize pups which are too weakened for rehabilitation.

## Material and methods

Stranded and/or abandoned pinnipeds found alive on German coasts (including islands) and sampled for this study were treated according to the “Directive for the treatment of sick, weakened or orphaned pinnipeds”. Only harbor seal pups with the prospect of survival were admitted to rehabilitation [28]. However, data of four of six pups which were not admitted and euthanized were included in this study as well.

Prior to the rehabilitation, abandoned harbor seal pups underwent a standardized clinical health check. This included visual inspection, check of reflexes, joints, umbilicus, respiratory and heart rate, determination of body temperature, reduced body length (axilla to tip of tail), axillary girth and weight which were also used to calculate a condition index. In addition, blood samples were taken for further investigations.

Data sets of 99 pups (95 that entered rehab and 4 that were euthanized prior to admission) were statistically evaluated. This included 53 females (52 which went to rehabilitation plus one euthanized pup) and 44 males (43 which went to rehabilitation and one euthanized pup), the gender of two euthanized animals was not determined. 69 of the admitted pups (31 males, 38 females) survived rehabilitation, while 14 female and 12 male pups died during rehabilitation, which shows an almost equal ratio of dead female and male pups.

### Differential hematology and serum chemistry

Venous whole blood from the fasting pups was drawn from the epidural vertebral vein with a needle (1.2 × 100 mm) and a syringe (10 ml). For differential hematology and serum chemistry (triglycerides and protein), venous whole blood was collected in tubes with ethylenediaminetetraacetic acid (EDTA) anticoagulant and tubes with coagulation gel for serum extraction, respectively. Tubes were kept at room temperature and analyzed or centrifuged and frozen within two hours. Differential hemogram profiles were generated with a ScilVet ABC™ Animal Blood Counter (Scil Animal Care Company GmbH, D-68519 Viernheim, Germany), calibrated for harbor seal blood samples including manual counts for leukocyte differentials. Serum separator tubes were centrifuged for 15 minutes after blood was clotted (Hettich™ EBA I centrifuge, Andreas Hettich GmbH & Co. KG, D-78532 Tuttlingen, Germany). Differential blood parameters included WBC (10^3^/μl), RBC (10^6^/μl), HGB (g/dl), HCT (%), thrombocytes (g/l), lymphocytes (%), monocytes (%), neutrophils (%), lymphocytes (10^3^/μl), monocytes (10^3^/μl) and neutrophils (10^3^/μl).

Serum was separated, extracted, kept frozen at −20°C and later sent to Synlab Vet in Geesthacht, Germany (an accredited veterinary laboratory) for the determination of total serum protein and triglyceride concentrations. For repeated measurements of total protein and triglyceride concentrations, blood was withdrawn from 12 fasting pups in the mornings additionally on day 10, day 20 and prior to release.

### Blood gas analysis

For the immediate use in blood gas analysis, 1 ml of venous whole blood was added to a microvette tube containing lithium heparin anticoagulant (Sarstedt® tubes, Sarstedt AG & Co., D-51582 Nümbrecht, Germany) and thoroughly inverted. Evacuated vacuum tubes were not recommended by the manufacturer of the blood gas analyzer because gases like CO_2_ dissolve faster into a vacuum than into air, leading to decreased results for pCO_2_, HCO_3_^–^ and TCO_2_[29,30].

Samples were analyzed for chemistry and blood gas concentrations no later than 10 minutes after blood withdrawal, using an i-STAT® 1 Portable Clinical Analyzer by Abbott (Abbott Point of Care Inc., Abbott Park, IL 60064, USA via Scil Animal Care Company GmbH, D-68519 Viernheim, Germany). The cartridge configuration “i-STAT® EC8+” used for this study contains the following variables: sodium (Na^+^), potassium (K^+^), chloride (Cl^-^), blood urea nitrogen (BUN), glucose (GLU) (all measured in mmol/l) and the blood gas variables pH (no unit), partial carbon dioxide pressure (pCO_2_ in mm Hg), plus the variables total carbon dioxide concentration (TCO_2_ in mmol/l), bicarbonate (HCO_3_^–^ in mmol/l), base excess of the extracellular fluid (BE^ecf^ in mmol/l) and anion gap (AnGap in mmol/l) that were calculated by the i-STAT® 1 analyzer on the basis of the measured variables.

Accuracy of the i-STAT® 1 analyzer was rated exact or at least tolerable [30,31], and lithium heparin is only known to influence values of calcium, which was not measured in this study [32].

Table 1 shows the number of blood gas datasets that were obtained from admitted pups of 2010.

**Table 1:** Number of available blood gas datasets from admitted pups of 2010

**Admitted pups in 2010 (total)**	**Admitted individuals sampled for blood gas analysis**	**Non-fasting individuals excluded from blood gas analysis**	**Sick individuals sampled, but not admitted (euthanized, N = 6)**	**Sum of obtained blood gas datasets**
**108**	95	13	4	**99**

### Statistical methods

Statistic evaluations were performed with the free statistic software *R* (R version 2.11.1 (2010-05-31) [33]. Measured values were mainly non-normally distributed. Generalized linear models for non-normally distributed values (glm, error structure: gamma family for continuous data) were applied for all data of blood gas variables to test for significant differences between genders and between survivors and non-survivors.

**Figure 1:**
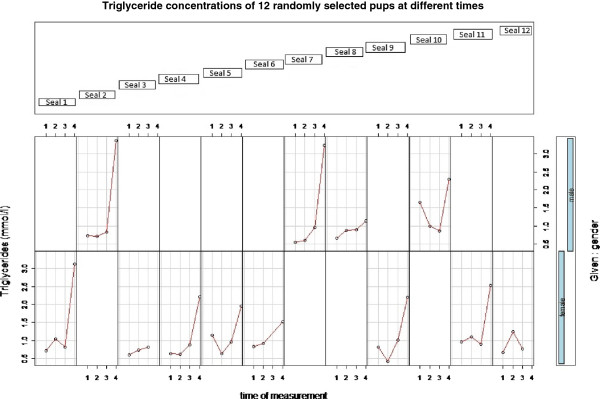
**Triglyceride concentrations in 12 pups at different times.** Top blue boxes: names. Upper row: plots of males. Lower row: plots of females. X-axis: time of measurement (1 = admission, 2 = 10th day, 3 = 20th day, 4 = pre-release). Y-axis: Values of triglycerides measured from serum in mmol/l.

The development of triglyceride and protein in serum samples of 12 pups with potential trends over time was calculated with a generalized linear mixed-effects model (lme4, error structure: quasi-Poisson) [34] and displayed in a co-plot (Figure 1).

Statistical significance was assumed whenever p < 0.05, slight significance when p > 0.05 and ≤ 0.07. Linear regression analysis was performed to detect potential correlations between certain blood gas variables (correlation assumed if R^2^ ≥ 0.75) [35].

## Results

### Medians and ranges of hematologic variables

Medians, ranges (minimum and maximum) and percentile ranges (5-95%) values of differential hemogram profiles, serum chemistry and blood gas parameters are shown in Table 2. Medians and ranges were preferred to arithmetic means and standard deviations because hematologic variables are generally not normally distributed.

**Table 2:** Median, percentile range (5 - 95%), range (minimum and maximum) and sample size (N) of differential hemogram profile, serum chemistry and blood gas analysis of blood from the epidural vertebral vein of harbor seal pups 2010 on admission to the Seal Center Friedrichskoog, Germany

**Variable**	**Median**	**Percentile range**	**Range**	**N**
*Hematology profile*				
WBC (10^3^/μl)	7.8	4.3 - 15.5	3.6 - 17.3	**99**
RBC (10^6^/μl)	5.6	4.5 - 6.6	4.5 - 7.0	**99**
HGB (g/dl)	20	14.7 - 23.9	12.7 - 24.9	**97**
HCT (%)	56.5	39 - 69	30 - 69.6	**98**
Thrombocytes (g/l)	346	88 - 558	19 - 598	**99**
Lymphocytes (%)	27.1	11.6 - 46.1	8.7 - 54.6	**99**
Monocytes (%)	4.4	2.5 - 7.4	1.8 - 8.0	**99**
Neutrophils (%)	68.3	46.5 - 85.2	18.5 - 88.0	**99**
Lymphocytes (10^3^/μl)	2	0.9 - 4.3	0.5 - 22.2	**99**
Monocytes (10^3^/μl)	0.3	0.1 - 0.75	0.0 - 0.9	**99**
Neutrophils (10^3^/μl)	5.3	2.7 - 11.5	2.4 - 15.2	**99**
*Serum chemistry*				
Glucose (mmol/l)	6.9	4.1 - 9.9	3 - 10.8	99
BUN (mmol/l)	7.3	4 - 12.6	3 - 14.7	99
Sodium (mmol/l)	140	136 - 144	135 - 147	99
Potassium (mmol/l)	3.9	3.3 - 4.8	2.7 - 5.2	99
Chloride (mmol/l)	105	99 - 111	97 - 113	99
Total Protein (g/dl)	6.7	5.4 - 7.6	3.8 - 8.0	96
Triglycerides (mmol/l)	0.9	0.5 - 1.7	0.2 - 1.9	95
*Blood gas analysis*				
TCO_2_ (mmol/l)	33	28 - 39.5	27 - 47	99
pH	7.44	7.38 - 7.52	7.36 - 7.55	99
pCO_2_ (mm Hg)	46.1	36.4 - 59.3	32.4 - 65.4	99
HCO_3_^–^ (mmol/l)	31.3	26.6 - 37.6	25.4 - 45.5	99
BE^ecf^ (mmol/l)	7	2 - 13	1 - 22	99
Anion Gap (mmol/l)	7	0.45 – 11.5	(− 2) - 15	99

WBC = white blood cell counts, RBC = red blood cell counts, HGB = hemoglobin, HCT = hematocrit, BUN = blood urea nitrogen, TCO_2_ = total CO_2_, pCO_2_ = partial CO_2_ pressure, BE^ecf^ = base excess of extracellular fluid.

Statistical test results for differences between genders and between survivors and non-survivors are shown in Table 3. Males were significantly heavier (0.6 kg) and had a slightly longer reduced length (1.5 cm) than females. Furthermore, females had significantly higher WBC, neutrophils (%) and neutrophils (10^3^/μl). Males had significantly higher lymphocytes (%) and monocytes (%). However, the female-to-male ratio of dead pups was even, suggesting that these variables did not affect survival.

**Table 3:** Distribution of hematologic variables (Shapiro-Wilk test, 95% confidence interval), ANOVA of genders and between survivors and non-survivors with generalized linear models (95% confidence interval)

**Variable**	**Normal distribution**	**Difference gender p**	**Difference (non)-survival p**
WBC (10^3^/μl)	–	0.006***^f^	0.23
RBC (10^6^/μl)	+	0.07*^f^	0.75
HGB (g/dl)	–	0.77	0.51
HCT (%)	–	0.67	0.66
Thrombocytes (g/l)	+	0.60	0.82
Lymphocytes (%)	+	0.003***^m^	0.26
Monocytes (%)	+	0.006***^m^	0.30
Neutrophils (%)	–	0.007***^f^	0.93
Lymphocytes (10^3^/μl)	–	0.19	0.15
Monocytes (10^3^/μl)	–	0.87	0.16
Neutrophils (10^3^/μl)	–	0.003***^f^	0.63
Glucose (mmol/l)	+	0.43	0.22
BUN (mmol/l)	–	0.31	0.22
Sodium (mmol/l)	–	0.41	0.78
Potassium (mmol/l)	+	0.51	0.46
Chloride (mmol/l)	–	0.21	0.09*^s^
Total Protein (g/dl)	–	0.42	0.75
Triglycerides (mmol/l)	–	0.11	0.60
TCO_2_ (mmol/l)	–	0.22	0.79
pH	+	0.49	0.31
pCO_2_ (mm Hg)	–	0.14	0.40
HCO_3_^–^ (mmol/l)	–	0.24	0.84
BE^ecf^ (mmol/l)	–	0.23	0.95
Anion Gap (mmol/l)	–	0.41	0.19

+ = normal distribution, - = non-normal distribution, *** = significant difference, * = slight difference, m = higher values in males, f = higher values in females, s = higher values in survivors.

The distribution of age at death for 22 non-survivors (four values missing, because no age determination was performed) showed that the majority of the non-survivors (15 pups) died between day 10 and day 20 after their estimated birth (median = day 17).

### Trends in variation of serum triglyceride and total protein concentrations

Twelve pups (four males, eight females) were sampled repeatedly (on admission, day 10, day 20 and prior to release). Serum triglyceride and total protein concentrations from fasting serum were then analyzed in order to display the development of concentration over time. The analysis showed that there was a positive significant (p = 0.002) influence of time on the development of serum triglycerides from the first sampling to the last (pre-release). No such trend was detected for serum protein though (Figure 1).

### Potential correlations between blood gas variables

Linear regression analysis revealed no significant correlations between any of the measured variables with the exception of default positive variables. These are RBC and hemoglobin as well as default correlations between some blood gas variables that were measured by the i-STAT® 1 analyzer device and others that are only calculated by the device by using variables that were actually measured: The values for TCO_2_, HCO_3_^–^ and BE^ecf^ are always calculated by the device by using the measured values of pH and pCO_2_, so that certain parameters correlate by default.

## Discussion

### Differential hematology

The resulting median value for RBC of 5.6 × 10^6^/μl in this study seemed reasonable considering that RBCs are generally higher in neonatal harbor seals, slowly decreasing with age and first diving attempt, with a simultaneous rise of mean corpuscular volume for a higher oxygen-carrying capacity [7,32,36-39]. Increased RBC-levels are also associated with dehydration which is an additional cause in the present study [7,32].

Hemoglobin concentrations of pups on admission had a median value of 20 mg/dl, which is comparably higher than the median of 16.5 mg/dl in the study of Hasselmeier *et al.*[7], obtained from pre-release pups. Lander *et al.*[19] also found hemoglobin, hematocrit and red blood cell counts to decrease significantly during captivity for rehabilitation purposes.

The median value for the hematocrit of 56.5% was comparably higher than in the study of Hasselmeier *et al.*[7] with 46% for pre-release pups, which indicates that pups on admission were often dehydrated. Dehydration was treated with *Lactated Ringer’s Solution* containing Na^+^ Cl^-^ and glucose (5%).

Pups had a relatively low median value of 7.8 × 10^3^/μl WBCs, which indicates that the immune system of most harbor seal pups is still impaired shortly post-parturition [40-42], whereas pre-release pups in the study of Hasselmeier *et al.*[8] displayed median values of 9.0 × 10^3^/μl of WBCs. During rehabilitation, pups often show WBC’s exceeding 12 × 10^3^/μl, indicating leukocytosis due to stress or infection (normal range: 7 to 9 × 10^3^/μl; [23]). Other possible causes for decreased WBC numbers are malnutrition or overwhelming infection. However, stress and inflammation also often result in monocytoses with simultaneous decrease of lymphocytes and eosinophils [32].

Leukocytes are sensitive to physiological changes in connection with stress, bacteria and viruses [7]. Elevated levels can indicate inflammation, tumors or infection [32]. However, WBCs in this study were not significantly different between survivors and non-survivors.

Reported ranges of blood variables can only be guidelines. Numerous parameters have to be considered when comparing ranges of blood variables in harbor seals, such as age, sex, health status, season, nutrition, location and living conditions. The comparison of harbor seals from the German North Sea grouped by age, season and living conditions (permanently captive, free-ranging and rehabilitated) has shown significant differences in hematologic profiles [7].

### Serum chemistry and blood gases

The use of a portable i-STAT® 1 analyzer and heparinized whole blood for the determination of serum chemistry variables in harbor seals was not previously performed for such a large sample size (N = 99). When comparing serum chemistry values of other studies, one should consider that they were determined with special chemical analyzers and serum as a medium. Accuracy of the i-STAT® 1 analyzer was rated exact or at least tolerable [30,31], and lithium heparin is only known to influence values of calcium, which was not measured in this study [32].

Five pups of this study were hypoglycemic (values < 4.4 mmol/l, normal: 5.5 mmol/l; [23]). Especially newborn harbor seals are known to suffer from hypoglycemia, indicating systemic disease, malnutrition, starvation or hepatic disease [32]. However, they survived rehabilitation which supports the theory of Greig *et al.*[43] and Marrie & Gaydos [44] that most serum chemistry variables are not predictive for rehabilitation outcome and/or survival of harbor seal pups.

The same applies to BUN (blood urea nitrogen), which Roletto [45] reported to be higher in diseased, than in clinically healthy pups. However, values of surviving and non-surviving pups of this study did not differ significantly. Values are also similar to data reported by Morgan *et al.*[25].

Values for sodium (median: 140 mmol/l, range: 135 – 147 mmol/l) were considered to be within normal ranges derived from marine mammal medicine [45]. Potassium values mostly varied within normal ranges. Values below 3.5 mmol/l were interpreted as a deficiency and were compensated with K^+^ Cl^-^ solution administered intravenously [18,32].

When comparing chloride values with those obtained in other studies (e.g. [25,32]) it appears that this variable remains fairly stable.

Total serum protein concentrations on admission (percentile range of 5.4 to 7.6 g/dl) were slightly lower than those reported by Dierauf & Gulland [32] for 42 rehabilitated weanlings. This indicates that total protein is increasing during rehabilitation. However, there was no indication of that in the ANOVA performed on data from twelve repeatedly sampled pups. This may be influenced not only by the protein uptake from artificial milk replacers but also by shifts in protein concentrations of the subsequent herring diet during rehabilitation [32]. Roletto [45] reported significantly lower values in pups suffering from emaciation, respiratory and heart failure, enteritis and liver failure compared to clinically healthy ones. It is questionable, though, whether a comparison with other studies is reasonable because nutritional compositions of feeding formulas may vary [32].

Similar facts apply to triglyceride concentrations: They can vary significantly with diet (in particular seasonal caloric value of prey and time of last food intake) among different populations and even between individuals [22]. This makes the establishment of reference ranges nearly impossible, if not unreasonable, as Roletto [45] did not find significant differences in values between clinically healthy and sick pups, but there were strong variations in individual values. However, ranges of values obtained in our study were almost identical with clinically healthy pups in Roletto [45], revealing no influence on survival chances.

Furthermore, an ANOVA revealed a significant positive influence of time on changes of triglyceride concentrations (Figure 1). An increase of triglyceride concentrations during rehabilitation was also observed by Greig *et al.*[43].

The median pH value of 7.44 measured in this study conforms to the mammalian norm of 7.4 during non-diving periods. During long dives the pH in marine mammals can shift to as low as 6.8 [11]. The same applies to the median pCO_2_ value of 46.1 mm Hg, which is virtually identical to the mammalian norm value of 46 mm Hg in venous blood [10]. However, the range of values for pCO_2_ was quite large, but there were no significant differences between survivors and non-survivors. There was no negative correlation between pCO_2_ and pH values (R^2^ = 0.42, significance only when R^2^ ≥ 0.75), suggesting that high pCO_2_ values do not necessarily occur with low pH values and respiratory acidosis but could also be involved in secondary mechanisms compensating metabolic alkalosis. Therefore the application of nomograms [15,16] is useful.

To understand acid–base disorders, it is essential to monitor the overall situation of an individual repeatedly over a longer period of time to detect the respective primary disease which might have caused the acid–base disorder. Therefore, it is important to determine the buffering capacity of the blood through the HCO_3_^–^ concentration. Obtained values of this study are comparable to those of cross-bred calves and Hawaiian monk seals *Monachus schauinslandi*[27,46]. With the base excess values always positive, there is no need for the assumption that buffering capacities were too low. Some rather high values might be explained by the condition of pups at the time of admission to rehabilitation; gastrointestinal distress (vomiting and diarrhea) could have led to a loss of electrolytes and thus might have caused temporary metabolic alkalosis [17].

The normal range for the anion gap in humans is 10–12 mmol/l, which is a bit higher than the median value of 7 mmol/l in this study, but lower anion gap values are not associated with any pathological condition [47].

Measured blood gas values of this study can hardly be compared to other studies (e.g. [25-27]) on blood gases in pinnipeds. Either, no complete sets of variables were measured, or other measurement techniques were used (including different analyzers or sampling techniques, where gases can dissolve and alter results). The latter might also explain why our TCO_2_ values were generally higher than those in other studies. Values are also higher than those of a study on venous blood gases of dogs by Ilkiw *et al.*[48] but similar to those measured in cross-bred calves by Gunes & Atalan [46] (both arterial and venous pH values). This finding suggests that results may vary among different studies, but are comparable to other marine and terrestrial mammal species.

### Predictors of survival

Percentile ranges of pups’ weights (7.6 - 12.5 kg) are similar to values given in common literature, such as Jefferson *et al.*[49] or Burns [2]. Reduced length was slightly higher in males than in females and also slightly higher in survivors than in non-survivors.

Physical variables can help to determine survival chances because low admission weights, prior trauma and prematurity are mentioned repeatedly in connection with higher mortality in harbor seal pups during rehabilitation [22,44,50]. The pups classified as premature in the present study (N = 3) belonged to the group of survivors, and weights of survivors were only slightly higher than those of non-survivors. Outcomes of a study conducted on 102 harbor seal pups by Marrie & Gaydos [44] suggest that a high weight-to-length ratio significantly increases the probability of successful rehabilitation. Different approaches including axillary girth were applied by Trumble & Castellini [21] and Lander *et al.*[19].

However, the use of such data for estimations of survival chances should be made with care because they yield a prognosis rather than a prediction of survival or death.

## Conclusions

No significant differences were found in physical or hematologic variables between survivors and non-survivors. Clinical chemistry and blood variables were not associated with survival or rehabilitation success. Simultaneously, different feeding strategies with different milk replacer formulas and/or different weaning times could be tested in different control groups. This investigation makes no claim of being complete but it can serve as a stepping stone towards the establishment of reference ranges for poorly investigated variables, such as pH and its derivatives, in harbor seal pups. The weight-to-length ratio is suggested to be a good indicator for survival chances, but needs to be combined with statistical evaluations.

## Abbreviations

AnGap: Anion gap; BE^ecf^: Base excess of the extracellular fluid; BUN: Blood urea nitrogen; EDTA: Ethylenediaminetetraacetic acid; GLU: Glucose; HCT: Hematocrit; HGB: Hemoglobin; pCO_2_: Partial carbon dioxide pressure; RBC: Red blood cell counts; TCO_2_: Total carbon dioxide concentration; WBC: White blood cell counts.

## Competing interests

All authors declare that they have no competing interests.

## Authors’ contributions

KW, JD and TR collected samples and data. KW and JD carried out blood gas analyses. Data treatment was conducted by KW, JD and US. SA performed statistical analysis. KW, JD, SA and US drafted the manuscript. All authors read and approved the manuscript.

## References

[B1] Siebert U Müller S Gilles A Sundermeyer J Narberhaus I Narberhaus I, Krause J, Bernitt U Artensteckbriefe Marine Säugetiere Bedrohte Biodiversität in der deutschen Nord- und Ostsee. Empfindlichkeiten gegenüber anthropogenen Nutzungen und den Effekten des Klimawandels 2012 Bonn, Germany: Bundesamt für Naturschutz (BfN) 518 527

[B2] Burns JJ Perrin WF, Würsig B, Thewissen JGM Harbor seal and spotted seal Encyclopedia of Marine Mammals 2009 2 San Diego, CA: Academic, Press 533 542

[B3] Reijnders PJH Brasseur SMJM Borchardt T Camphuysen K Czeck R Gilles A Jensen LF Leopold M Lucke K Ramdohr S Scheidat M Siebert U Teilmann J Marine mammals Thematic Report No. 20 Wadden Sea Ecosystem No. 25 2009 Wilhelmshaven, Germany: CWSS; 2009: Chapter Marine Mammals 1 16

[B4] Bowen WD Beck CA Austin DA Perrin WF, Würsig B, Thewissen JGM Pinniped ecology Encyclopedia of Marine Mammals 2009 2 San Diego, CA: Academic, Press 852 861

[B5] Brasseur SMJM Fedak MA CWSS Habitat use of harbour seals in relation to recreation, fisheries, and large infra-structural works Proceedings of the International Symposium at EcoMare, 29–30 November 2002; Texel, The Netherlands. CWSS: Management of North Sea Harbour and Grey Seal Populations Wadden Sea Ecosystem No. 17 2003 Wilhelmshaven, Germany: Common Wadden Sea Secretariat (CWSS) 27 31

[B6] Hasselmeier I Evaluation of blood tests to assess the health status of harbor seal (*Phoca vitulina vitulina*) of the German North Sea Phd thesis Berichte, Forschungs- und Technologiezentrum Westküste der Universität Kiel 42 2006 Büsum, Germany: University Kiel

[B7] Hasselmeier I Fonfara S Driver J Siebert U Differential hematology profiles of free-ranging, rehabilitated, and captive harbor seals (*Phoca vitulina*) of the German North Sea Aqua Mamm 2008 56 149 156 10.1578/AM.34.2.2008.149

[B8] Depocas F Hart JS Fisher HD Sea water drinking and water flux in starved and in fed harbor seals, *Phoca vitulina* Can J Physiol Pharm 1971 56 53 62 10.1139/y71-007 5572418

[B9] Heldmaier G Neuweiler G Vergleichende Tierphysiologie Vegetative Physiologie 2004 56 Berlin & Heidelberg, Germany: Springer Verlag

[B10] Schmidt-Nielsen K Animal physiology. Adaptation and environment 1997 5 Cambridge, MA: CambridgeUniversity Press

[B11] Elsner R Reynolds JE, Rommel SA Living in water. Solutions to physiological problems Biology of marine mammals 1999 Washington, DC: Smithsonian Institution Press 73 116

[B12] Kooyman GL Castellini MA Davis RW Physiology of diving in marine mammals Annu Rev Plant Physiol Plant Mol Biol 1981 56 343 356 10.1146/annurev.ph.43.030181.002015 7011189

[B13] Lenfant C Johansen K Torrance JD Gas transport and oxygen storage capacity in some pinnipeds and the sea otter Resp Physiol 1970 56 277 286 10.1016/0034-5687(70)90076-9 5445188

[B14] Irizarry R Reiss A Arterial and venous blood gases: indications, interpretations, and clinical applications Compendium: Continuing Education for Veterinarians 2009 56 1 7 20180211

[B15] Mikulcik P Rapidanalyse – Blutgase und mehr 2005 Siemensstrasse 3, 35463 Fernwald, Germany: Bayer Vital GmbH Diagnostics [http://www.medical.siemens.com/siemens/en_GLOBAL/gg_diag_FBAs/files/services/patientinformation/blutgasfibel_060605.pdf]

[B16] Siggaard-Andersen O The oxygen status algorithm. A computer program for calculation and graphical interpretation of the acid–base and oxygen status of the blood Version of 2005 with new temperature corrections for pH and pCO_2_ [http://www.siggaard-andersen.dk/]

[B17] Boemke W Krebs MO Rossaint R Blutgasanalyse Der Anästhesist 2004 56 471 494 10.1007/s00101-004-0680-6 15222335

[B18] Schneeweiss B Funk GC Reuter P Akute Störungen des Wasser-, Elektrolyt- und Säure-Basen-Haushaltes Springer Lexikon Diagnose und Therapie 2006 1 Heidelberg, Germany: Springer Verlag 1387 1404

[B19] Lander ME Harvey JT Gulland FMD Hematology and serum chemistry comparisons between free-ranging and rehabilitated harbor seal (*Phoca vitulina richardsi*) pups J Wildlife Dis 2003 56 600 609 10.7589/0090-3558-39.3.600 14567222

[B20] Hall AJ McConnell BJ Barker RJ The effect of total immunoglobulin levels, mass and condition on the first-year survival of grey seal pups FunctEcol 2002 56 462 474

[B21] Trumble SJ Castellini MA Blood chemistry, hematology, and morphology of wild harbor seal pups in Alaska J Wildlife Manage 2002 56 1197 1207 10.2307/3802953

[B22] Dierauf LA Dougherty SA Lowenstine LJ Survival versus non-survival determinants for neonatal harbor seals J Am Vet Med Ass 1986 56 1024 1028 3505918

[B23] Hall AJ McConnell BJ Barker RJ Factors affecting first-year survival in grey seals and their implications for life history strategy J Anim Ecol 2001 56 138 149 10.1046/j.1365-2656.2001.00468.x

[B24] Harding KC Fujiwara M Axberg Y Karkonen T Mass-dependent energetics and survival in harbour seal pups Funct Ecol 2005 56 129 135 10.1111/j.0269-8463.2005.00945.x

[B25] Morgan LW Jakush JL Simpson A Norman MM Pabst DA Simmons S Evaluation of hematologic and biochemical values for convalescing seals from the coast of Maine J Zoo Wildlife Med 2009 56 421 429 10.1638/2007-0032.1 19746855

[B26] Boily F Beaudoin S Measures LN Hematology and serum chemistry of harp (*Phoca groenlandica*) and hooded seals (*Cystophora cristata*) during the breeding season, in the Gulf of St. Lawrence, Canada J Wildlife Dis 2006 56 115 132 10.7589/0090-3558-42.1.115 16699154

[B27] Reif JS Bachand A Aguirre AA Borjesson DL Kashinsky L Braun R Antonelis G Morphometry, hematology, and serum chemistry in the Hawaiian monk seal (*Monachus schauinslandi*) Mar Mamm Sci 2004 56 851 860 10.1111/j.1748-7692.2004.tb01196.x

[B28] Richtlinie zur Behandlung von erkrankt, geschwächt oder verlassen aufgefundenen Robben Ministerium für Umwelt, Natur und Forsten des Landes Schleswig-Holstein. 1997 Amtsblatt Schleswig-Holstein: 500 [http://www.gesetze-rechtsprechung.sh.juris.de/jportal/?quelle=jlink&query=vvsh-7921.2-0001&max=true&psml=bsshoprod.psml]

[B29] Abbott Point of Care Gesamtkohlendioxid/(TCO2) [http://www.i-stat.com/products/ctisheets/716661-02E.pdf]

[B30] Silverman SC Birks EK Evaluation of the i-STAT hand-held chemical analyser during treadmill and endurance exercise Equ Vet J 2002 56 551 554 10.1111/j.2042-3306.2002.tb05481.x 12405749

[B31] Schneider J Dudziak R Westphal K Vettermann J Der i-STAT Analyzer. Ein neues, tragbares Gerät zur Bestimmung des Hämatokrits, der Blutgase und Elektrolyte Der Anästhesist 1997 56 704 714 10.1007/s001010050457 9382209

[B32] Dierauf LA Gulland FMD CRC Handbook of Marine Mammal Medicine 2001 2 Boca Raton, FL: CRC Press

[B33] R: A language and environment for statistical computing R Development Core Team. 2010 Vienna, Austria: R Foundation for Statistical Computing [http://www.R-project.org] ISBN 3-900051-07-0

[B34] Bates D Maechler M Bolker B lme4: Linear mixed-effects models using S4 classes Rpackage version 0.999999-0 2012 [http://CRAN.R-project.org/package=lme4]

[B35] Crawley MJ The R Book 2007 Chichester: John Wiley & Sons, Ltd

[B36] Wickham LL Elsner R White FC Cornell LH Blood viscosity in phocid seals: possible adaptations to diving J Comp Physiol B 1989 56 153 158 10.1007/BF00691735 2760283

[B37] Roletto J Dougherty SA Hematologic changes in young growing phocids (*Phoca vitulina* and *Mirounga angustirostris*) Proc Int Ass Aquat Anim Med 1983 56 29

[B38] Geraci JR Functional hematology of the harp seal (*Pagophilus groenlandicus*) Physiol Zool 1971 56 162 170

[B39] Lenfant C Andersen HT Physiological properties of blood of marine mammals The Biology of Marine Mammals 1969 New York: Academic, Press 95 116

[B40] Ross PS de Swart RL Visser IKG Vedder LJ Murk W Bowen WD Osterhaus ADME Relative immunocompetence of the newborn harbor seal, *Phoca vitulina* Vet Immunol Immunop 1994 56 331 348 10.1016/0165-2427(94)90077-9 7810064

[B41] Bossart GD Dierauf LA Dierauf LA Marine mammal clinical laboratory medicine Handbook of Marine Mammal Medicine: Health, Disease and Rehabilitation 1990 Boca Raton, FL: CRC Press 1 52

[B42] Ross PS Pohajdak B Bowen WD Addison RF Immune function in free-ranging harbor seal (*Phoca vitulina*) mothers and their pups during lactation J Wildlife Dis 1993 56 21 29 10.7589/0090-3558-29.1.21 8445787

[B43] Greig DJ Gulland FMD Rios CA Hall AJ Hematology and serum chemistry of stranded harbor seals in central California: Reference intervals, predictors of survival, and parameters affecting blood variables J Wildlife Dis 2010 56 1172 1184 10.7589/0090-3558-46.4.1172 20966268

[B44] Marrie K Gaydos J Determining risk factors associated with mortality of stranded harbor seals (*Phoca vitulina*) during rehabilitation Proc Intern Assoc Aqua Ani Med 2007 56 144

[B45] Roletto J Hematology and serum chemistry values for clinically healthy and sick pinnipeds J Zoo Wildlife Med 1993 56 145 157

[B46] Gunes V Atalan G Comparison of ventral coccygeal arterial and jugular venous blood samples for pH, pCO_2_, HCO_3_^–^, BE_ecf_ and ctCO_2_ values in calves with pulmonary diseases. Res Vet Sci 2006 56 148 151 10.1016/j.rvsc.2005.10.003 16376395

[B47] Stockham SL Scott MA Stockham SL, Scott MA Monovalent electrolytes and osmolality Fundamentals of Veterinary Clinical Pathology 2002 Iowa State: University Press, Blackwell Publishing 337 380

[B48] Ilkiw JE Rose RJ Martin ICA A comparison of simultaneously collected arterial, mixed venous, jugular venous and cephalic venous blood samples in the assessment of blood-gas and acid–base status in the dog J Vet Int Med 1991 56 294 298 10.1111/j.1939-1676.1991.tb03136.x 1748981

[B49] Jefferson TA Webber MA Pitman RL Marine Mammals of the World. A Comprehensive Guide to their Identification 2008 London: Academic Press/Elsevier

[B50] Dougherty SA Dierauf LA Clinical and laboratory predictors of survival in neonatal harbor seals, Phoca vitulina richardsi Proc Int Ass Aquat Anim Med 1983 56 25

